# Exploring the potential common denominator pathogenesis of system lupus erythematosus with COVID-19 based on comprehensive bioinformatics analysis

**DOI:** 10.3389/fimmu.2023.1179664

**Published:** 2023-06-22

**Authors:** Huiqiong Zeng, Yu Zhuang, Xiaojuan Li, Zhihua Yin, Xia Huang, Haiyan Peng

**Affiliations:** ^1^Department of Rheumatology, Shenzhen Futian Hospital for Rheumatic Diseases, Futian District, Shenzhen, Guangdong, China; ^2^Department of Rheumatology and Immunology, Huizhou Central People’s Hospital, Huizhou, Guangdong, China; ^3^Department of Public Health, Shenzhen Hospital of Southern Medical University, Shenzhen, China; ^4^Department of Xi Yuan Community Health Service Center, The Eighth Affiliated Hospital of Sun Yat-sen University, Shenzhen, Guangdong, China

**Keywords:** bioinformatics, system lupus erythematosus, COVID-19, diagnostic biomarkers, immune cells infiltration

## Abstract

**Objective:**

Evidences show that there may be a link between SLE and COVID-19. The purpose of this study is to screen out the diagnostic biomarkers of systemic lupus erythematosus (SLE) with COVID-19 and explore the possible related mechanisms by the bioinformatics approach.

**Methods:**

SLE and COVID-19 datasets were extracted separately from the NCBI Gene Expression Omnibus (GEO) database. The limma package in *R* was used to obtain the differential genes (DEGs). The protein interaction network information (PPI) and core functional modules were constructed in the STRING database using Cytoscape software. The hub genes were identified by the Cytohubba plugin, and TF-gene together with TF-miRNA regulatory networks were constructed *via* utilizing the Networkanalyst platform. Subsequently, we generated subject operating characteristic curves (ROC) to verify the diagnostic capabilities of these hub genes to predict the risk of SLE with COVID-19 infection. Finally, a single-sample gene set enrichment (ssGSEA) algorithm was used to analyze immune cell infiltration.

**Results:**

A total of 6 common hub genes (*CDC6, PLCG1, KIF15, LCK, CDC25C*, and *RASGRP1*) were identified with high diagnostic validity. These gene functional enrichments were mainly involved in cell cycle, and inflammation-related pathways. Compared to the healthy controls, abnormal infiltration of immune cells was found in SLE and COVID-19, and the proportion of immune cells linked to the 6 hub genes.

**Conclusion:**

Our research logically identified 6 candidate hub genes that could predict SLE complicated with COVID-19. This work provides a foothold for further study of potential pathogenesis in SLE and COVID-19.

## Introduction

The disease caused by the novel coronavirus (SARS-CoV-2) is known as COVID-19 (1). COVID-19 has become a global pandemic disease and brought a tremendous impact around the world since 2019 and remains at high risk of transmission, with the ongoing emergence of SARS-CoV-2 variants leading to the recurrence of continued spreading in many countries (2). As of January 24, 2023, the latest updated data from the World Health Organization (WHO) reached 664,618,938 confirmed cases of COVID-19 infection, with 6,722,949 deaths worldwide (3). Nearly half of the patients hospitalized with severe COVID-19 develop one or more complications including cardiovascular manifestations, acute respiratory distress syndrome, liver injury, anemia, and brain fog (4).

Systemic lupus erythematosus (SLE) is a heterogeneous chronic autoimmune disease with varied clinical manifestations (5). The pathogenesis of SLE is still unclear, and there is a lack of biomarkers and specific and effective tailored treatments (6). The prevalence varies from approximately 50 to 100 cases per 100 000 people in China, and the risk of death for patients with SLE is still 2 times higher than that of the general population (7). The COVID-19 pathophysiology has uncovered that it could lead to the emergence or exacerbation of autoimmune diseases (8).

SLE and COVID-19 infection are the focus of widely discussed during the COVID-19 epidemic, these two diseases share certain similarities in pathogenesis (9). Patients with SLE are at increased risk of COVID-19 infection, and SLE patients may be at increased risk of adverse outcomes from treatment with anti-SARS-CoV-2 (10, 11). There are reports analyzing cases of the worsening clinical course of SLE or inducing new-onset SLE during COVID-19 infection (12, 13). However, a definitive explanation for the pathogenetic basis of co-morbidity between SLE and COVID-19 is still lacking. Although hypotheses regarding dysfunctional immune response related to cytokine dysregulation resulted in the loss of tolerance to auto-antigens are promising.

The goal of this study was to explore the co-pathogenesis of SLE and COVID-19. We identified hub genes associated with the pathogenesis of SLE combined with COVID-19, and analyzed their enriched functions by integrating bioinformatics approaches. We further constructed the TF-gene regulatory network and TF-miRNA regulatory network of the hub genes. Simultaneously, we performed immune cell infiltration analysis and mined hub genes related to therapeutic drug prediction. The hub genes between SLE and COVID-19 identified in this study are expected to provide new insights into the biological mechanisms of both diseases.

## Methods

### Data collection and source

**Figure 1:**
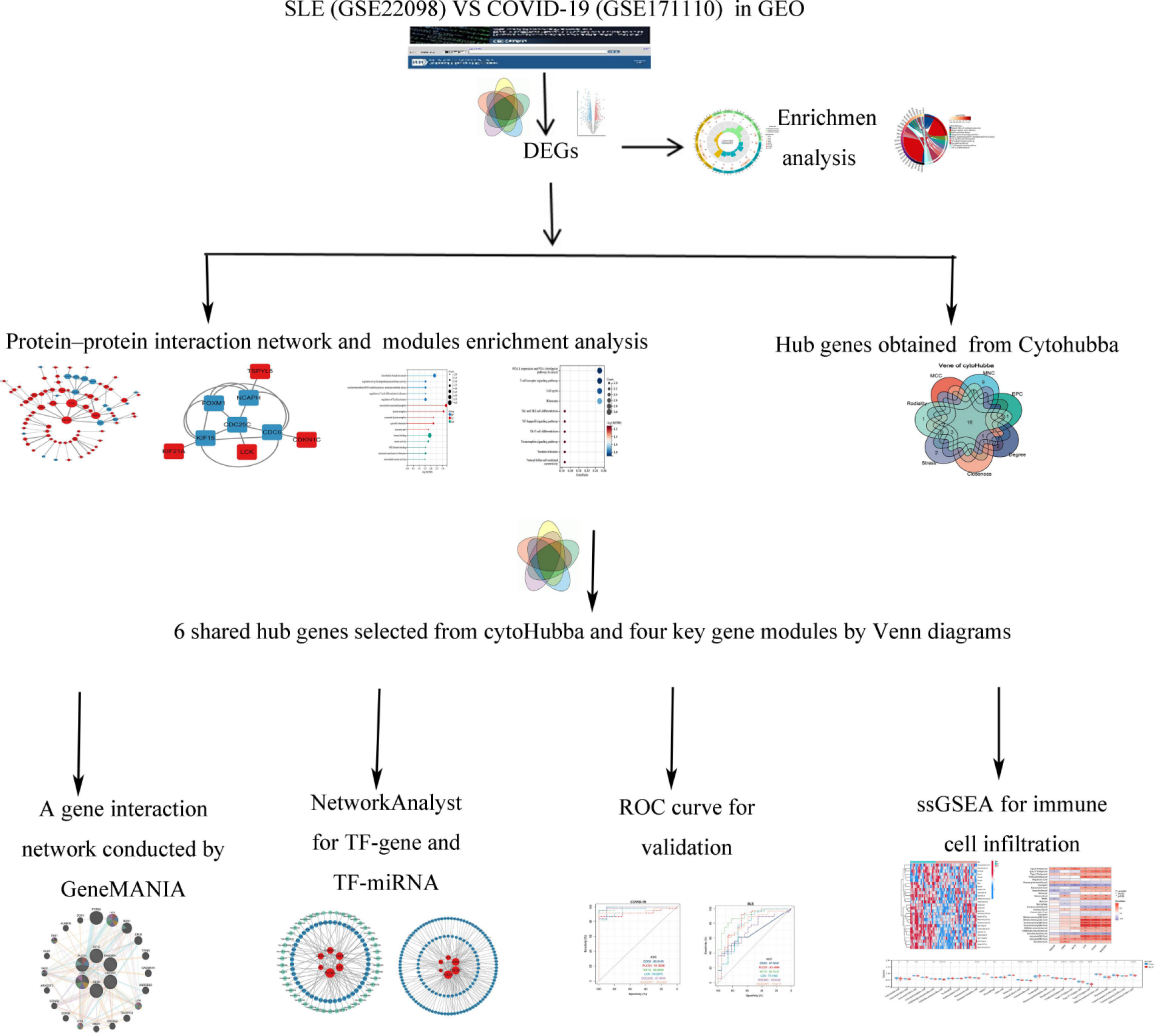
Study flow. The symbol * represents *P* < 0.05; ** represents *P* < 0.01; *** represents *P* < 0.001.

The SLE dataset (GSE22098) based on the Illumina HumanHT-12 V3.0 expression bead chip is downloaded from GEO (Gene Expression Omnibus, http://www.ncbi.nlm.nih.gov/geo/) database, and includes information from 28 SLE and 80 healthy control individuals with whole blood samples. The COVID-19 dataset (GSE171110) contained data on whole-blood gene expression profiles of 44 COVID-19 infection patients and 10 healthy controls, which used high throughput sequencing technology based on the Illumina HiSeq 2500 (Homo sapiens) platform (See **Figure 1** for the study flow).

### Differential expression genes identification between SLE and COVID-19

We used and normalized the data by implementing the *R* 3.6.1 software package “Limma”, and to explore the expression of differential genes (DEGs) in GSE171110 and GSE22098 between disease groups and the healthy controls samples, respectively (14, 15). Genes with adjusted *P*-value less than 0.05 and |log2-fold change (log2FC)| more than 1.0 were defined as statistically DEGs. The volcano plots of shared DEGs for COVID-19 and SLE were depicted using the pheatmap and ggplot2 *R* packages. The VennDiagram *R* package was used to detect overlapped DEGs in the two datasets.

### Functional enrichment analysis

To further reveal the potential functions of the shared DEGs, enrichment analysis of the shared DEGs was illustrated by ClusterProfiler in *R*. Gene Ontology analysis (GO) was performed to identify the top six GO terms including molecular functions (MF), biological processes (BP), and cellular components (CC). The threshold set for the shared DEGs was considered statistically significant with a false discovery rate (FDR) of < 0.1 and a *P* value < 0.05.

### Construction of protein-protein interaction network and hub genes selection

Protein-protein Interaction (PPI) networks are essential for the development of functional biology research. It maps DEGs to PPI data from publicly available databases to identify the pathways in which DEGs are involved (16). Cytoscape is an open-source software for visualizing, analyzing, and modeling biological networks (17).

Using STRING (https://string-db.org) database (version 11.5), a PPI network for the shared DEGs was constructed. We carried out a co-expression network using the Cytoscape software (version 3.9.1). Cytoscape molecular complex detection (MCODE) is used to pick out PPI interacting sub-networks in shared DEGs. The default parameters are as follows: Degree Cutoff: 2, Node Score Cutoff: 0.2, K-Core: 2, Max Depth: 100.

The CytoHubba plugin algorithm is used to searched for hub genes in the PPI network through seven topological analysis algorithms (Closeness, MCC, Degree, MNC, Radiality, Stress, and EPC), which were visualized by Venndiagram *R* package. The online Hiplot platform (https://hiplot.com.cn/cloud-tool) was used to obtain co-hub genes between the candidate genes obtained from plug-in CytoHubba and MCODE. We incorporated the co-hub genes into the online tool GeneMANIA (http://genemania.org) to conduct a gene co-expression network, in which an analysis of genes interacting with the co-hub genes on COVID-19 and SLE and the gene set function predictions were carried out and visualized (18).

### Identification of relevant transcription factors and TF-miRNAs regulatory network

Transcription Factors (TFs) are proteins that bind DNA in a sequence-specific manner and regulate transcription. TFs can control chromatin and transcription by recognizing specific DNA sequences to form a complex system that directs genome expression (19). To explore the potential TFs which may regulate the hub genes, we used the NetworkAnalyst 3.0 online tool (https://www.networkanalyst.ca/) by H. sapiens to predict TFs through the ENCODE database which contains chip-seq data for many TFs and complete a TF-gene regulatory network map using Cytoscape (20). The TF-miRNA coregulation network was obtained using the RegNetwork web tool.

### Validation of shared DEGs on COVID-19 and SLE

The expression level and diagnostic value of the obtained hub genes were constructed by receiver operating characteristic (ROC) curves and the area under the curve (AUC) with 95% confidence intervals (CI) to assess the levels of hub genes distinguishing on COVID-19 and SLE using the qROC package in *R* software. The AUC parameter over 0.60 is defined as optimal shared diagnostic biomarkers for predicting SLE with COVID-19.

### Immune cell infiltration analysis

The box line plots visualized the 28 types of immune relative infiltration cells with the expression of GSE171110 and GSE22098 datasets by using the single-sample gene set enrichment analysis (ssGSEA) algorithm, the assembled gene set of 782 marker genes in 28 immune cell types was used to evaluate 28 immune cells’ infiltration levels based on reference gene set (21). Violin plots were visualized and correlated to screen the differential expression levels of the 28 immune infiltrating cells. Spearman correlation was used to perform the relationship between hub genes and infiltrating immune cells. The “ggplot2” package was employed to exhibit the results. *P*-value < 0.05 was confirmed to be statistically different.

The relative infiltration levels of 28 immune cells in the GSE171110 and GSE22098 datasets were quantified using the ssGSEA algorithm. Box plots were drawn to demonstrate the differential expression levels of the 28 immune infiltrating cells. Spearman’s correlation was calculated for the 28 immune infiltrating cells with central genes and then visualized using the ‘ggplot2’ package.

## Result

### Identification of differential expressed genes

**Figure 2:**
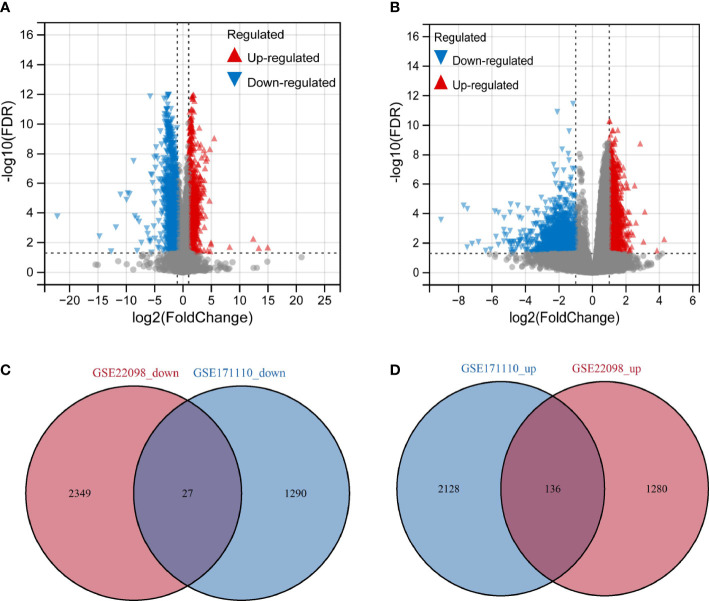
Volcano map of differentially expressed gene (DEGs) and shared gene identification **(A)** Represents DEGs from dataset GSE171110. **(B)** Represents DEGs from dataset GSE22098. Red represents up-regulated genes, green represents down-regulated genes, and grey represents genes that are not differentially expressed. **(C)** Venn’diagram of the down-regulated DEGs with GSE171110 and GSE22098 dataset. **(D)** Venn’diagram of the up-regulated DEGs with GSE171110 and GSE22098 dataset.

In our work, GSE171110 and GSE22098 were obtained from GEO database, and we uncovered the DEGs using the limma tool. The DEGs between SLE and COVID-19 selected datasets were input to VennDiagram *R* package for the identification of overlapped genes among these microarray datasets. A total of 3581 DEGs were obtained in COVID-19 dataset, including 2264 up-regulated genes and 1317 down-regulated genes (**Figure 2A**). As for the SLE dataset, we identified 3792 DEGs, including 1,416 upregulated genes and 2,376 downregulated genes (**Figure 2B**). By comparing these two datasets, we obtained 163 shared DEGs in SLE and COVID-19 total (**Figures 2C, D**). Of these common genes, 136 were up-regulated and 27 were down-regulated. The results suggest that there might have molecular similarity between COVID-19 and SLE.

### Functional enrichment analysis of gene set

To further explore the possible molecular mechanisms in SLE associated with COVID-19 co-morbidity, we integrated gene ontology (GO) and Kyoto Encyclopedia of Genes and Genomes (KEGG) pathway annotations as features to characterize 163 shared target genes obtained in the above screening step.

**Figure 3:**
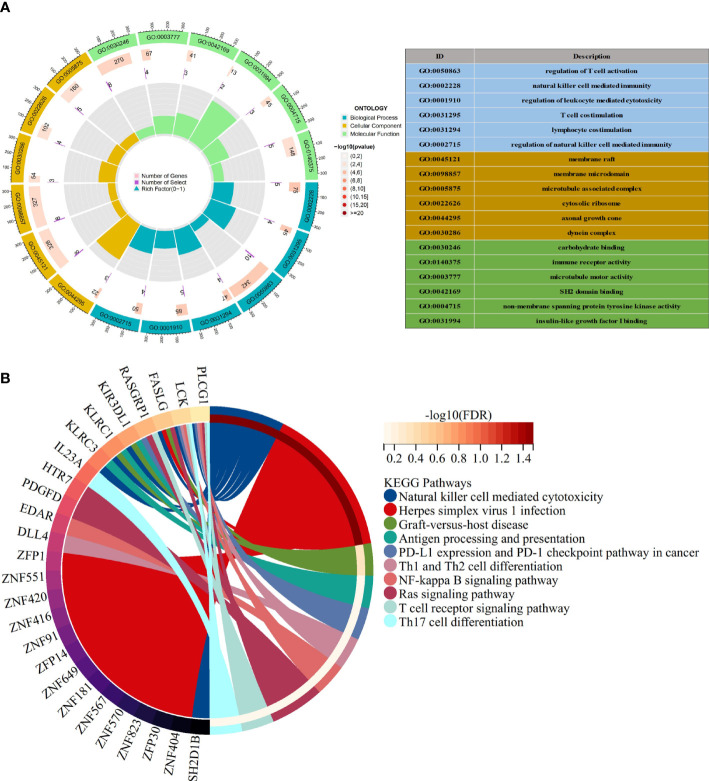
Functional enrichment analysis **(A)** The top 6 items of GO enrichment analysis. **(B)** The top 10 items of KEGG enrichment analysis.

Many of these GO items were associated with immunity, with the top 6 most significantly GO-enriched pathways (**Figure 3A**). Molecular function (MF) for shared genes analysis showed that immune receptor activity (GO: 0140375), and non-membrane spanning protein tyrosine kinase activity (GO: 0004715) were identified. As to cellular component (CC) ontology, these genes were mainly situated in the cytosolic ribosome (GO: 0022626). In the biological process (BP) category, the genes were primarily enriched in regulating T cell activation (GO: 0050863), natural killer cell-mediated immunity(GO: 0002228), and T cell co-stimulation (GO: 0031295). Pathways for the shared target genes were verified by KEGG enrichment analysis (**Figure 3B**). Genes in the KEGG category were enriched in the natural killer cell-mediated cytotoxicity signaling pathway, antigen processing, and presentation signaling pathway.

### Protein-protein interaction and gene modules analysis

**Figure 4:**
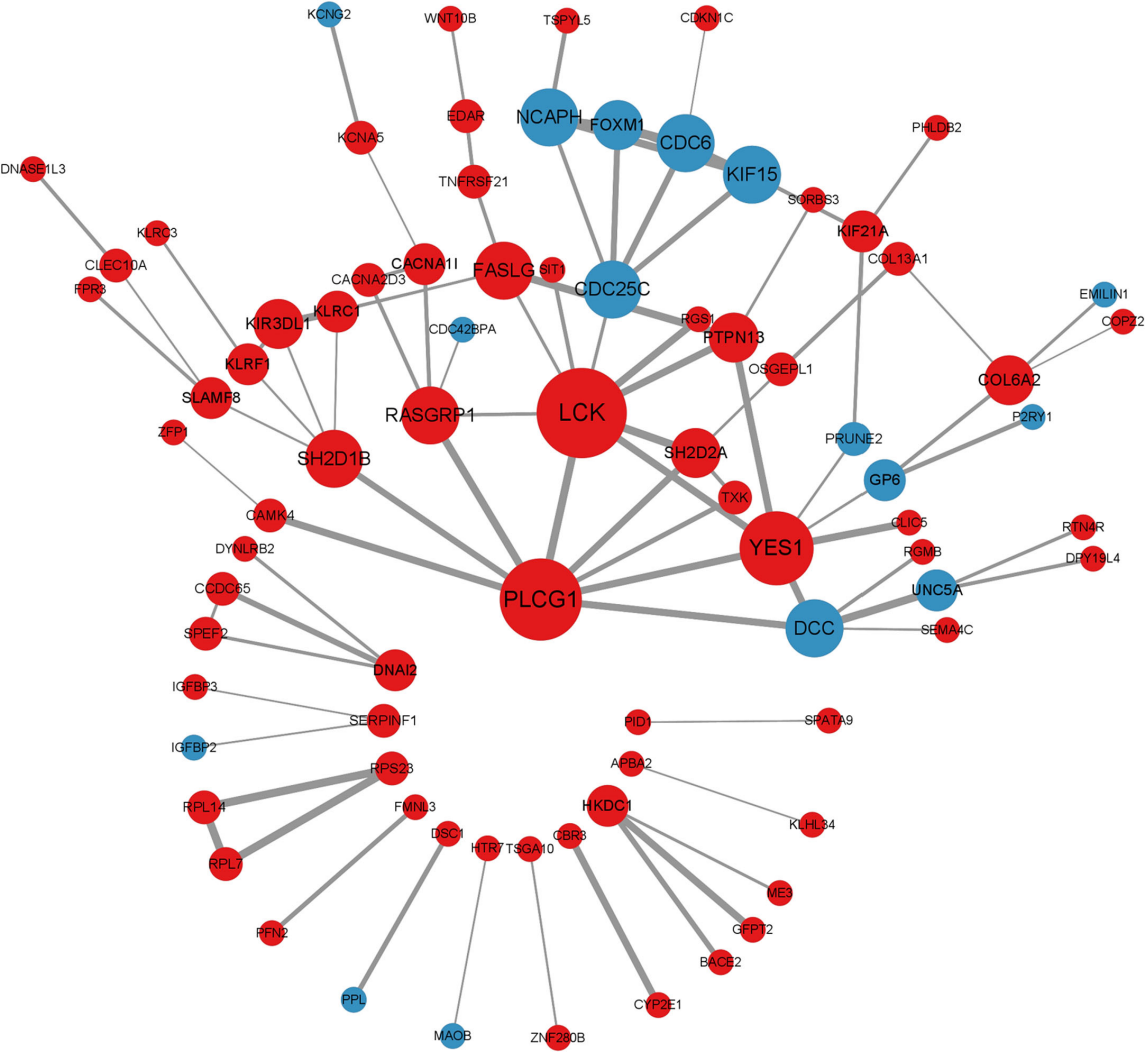
Visualization of the protein-protein interaction (PPI) network was constituted by STRING.

**Figure 5:**
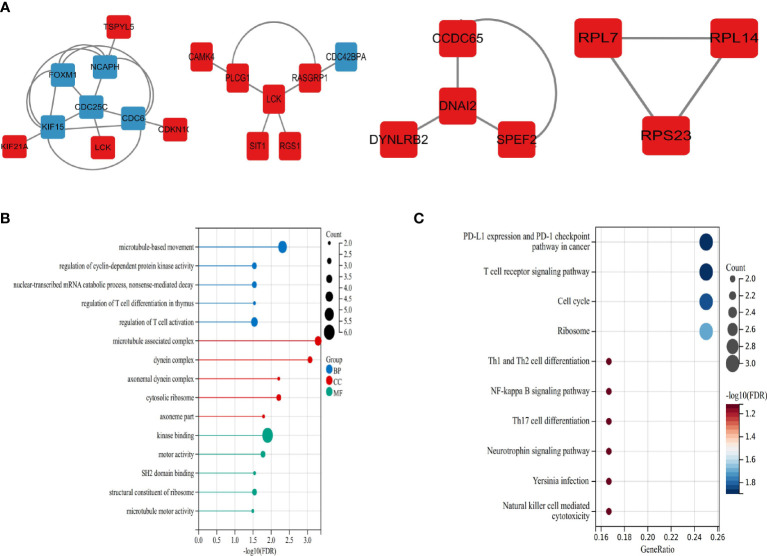
Significant modules and roles of PPI network **(A)** The top four key gene modules identified by MCODE plug-in of Cytoscape. **(B)** Bubble plots of Gene ontology (GO) enrichment for modules associated with the biological processes (GO-BP, blue), the cellular components (GO-CC, red), and the molecular functions (GOMF, green). **(C)** Bubble plots of Kyoto Encyclopedia of Genes and Genomes (KEGG) pathway analysis.

We mapped the shared target genes to the Protein-Protein Interaction (PPI) network for further exploring their potential interactions. This PPI contained a total of 82 nodes and 92 edges, in which the PPI interaction score is higher than 0.4 (**Figure 4**). the PPI network was constructed *via* the online search tool STRING. Hub gene modules were obtained using the Molecular Complex Detection (MCODE) plug-in in the Cytoscape tool, and four core modules including 22 shared DEGs were finally utilized (**Figure 5A**). Functional enrichment analysis revealed that these module genes were mainly enriched concerning regulation of T cell differentiation in the thymus, and regulation of T cell activation, microtubule-associated complexes, etc (**Figure 5B**). KEGG pathway analysis revealed that microtubule associated complex, kinase binding, nuclear-transcribed mRNA catabolic process, regulation of T cell differentiation in the thymus, and regulation of T cell activation signaling pathways were involved in these shared genes (**Figure 5C**).

### Identification of hub genes by MCODE and CytoHubba

**Figure 6:**
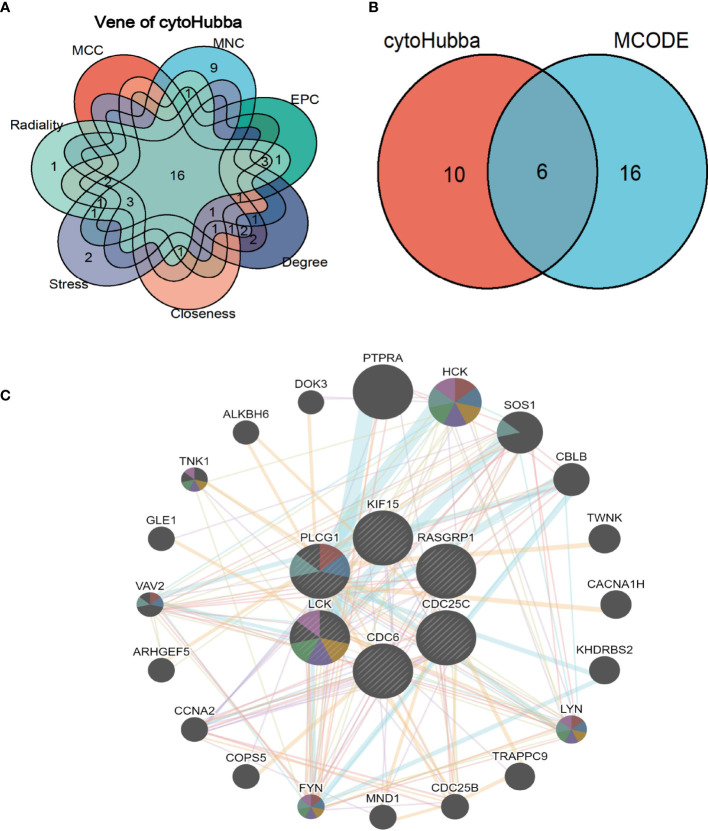
Shared hub genes identification and functional interactions network diagram **(A)** Venn diagram showing the identification of common hub genes by seven algorithms (Closeness, MCC, Degree, MNC, Radiality, Stress, and EPC) using the Cytohubba plug-in. **(B)** Venn diagram showing the 6 crossover hub genes between the candidate genes of CytoHubba and MCODE. **(C)** The GeneMANIA diagram shows the co-expression interactions between the 6 identified shared hub genes and their neighboring genes. Color codes indicate functions shared by genes.

CytoHubba plug-in in Cytoscape tool was integrated for topological analysis to identify hub genes using seven algorithms. Finally, a total of 16 common DEGs obtained from the intersection of CytoHubba were visualized through the intersection of Venn diagrams, including *YES1, SH2D2A, PTPN13, RASGRP1, PLCG1*, *SH2D1B, KIF15, KLRC1, KIR3DL1, LCK, KLRF1, CDC6, DCC, CACNA1I, FASLG*, and *CDC25C* (**Figure 6A**).

The intersection of CytoHubba candidate DEGs with the 22 shared DEGs in the four modules was then taken, and 6 shared genes were revealed overlapping and visualized by Venn diagram (**Figure 6B**). A GeneMANIA biological function analysis was adopted to investigate the genes with common properties and similar functions to the above 6 shared DEGs, as well as to demonstrate the interactive functional association network between genes. A total of 20 molecules were most associated with the 6 common DEGs. The results revealed that 44.33% co-expression between genes, physical interactions of 5.71%, co-localization of 8.85%, prediction of 20.30%, and pathway of 18.97% (**Figure 6C**). These genes functions were mainly associated with immune response-regulating cell surface receptor signaling pathway involved in phagocytosis, Fc receptor-mediated stimulatory signaling pathway, protein autophosphorylation, protein tyrosine kinase activity, cytoplasmic side of the plasma membrane, Fc receptor signaling pathway, and extrinsic component of plasma membrane.

### Construction of transcriptional level regulatory networks

**Figure 7:**
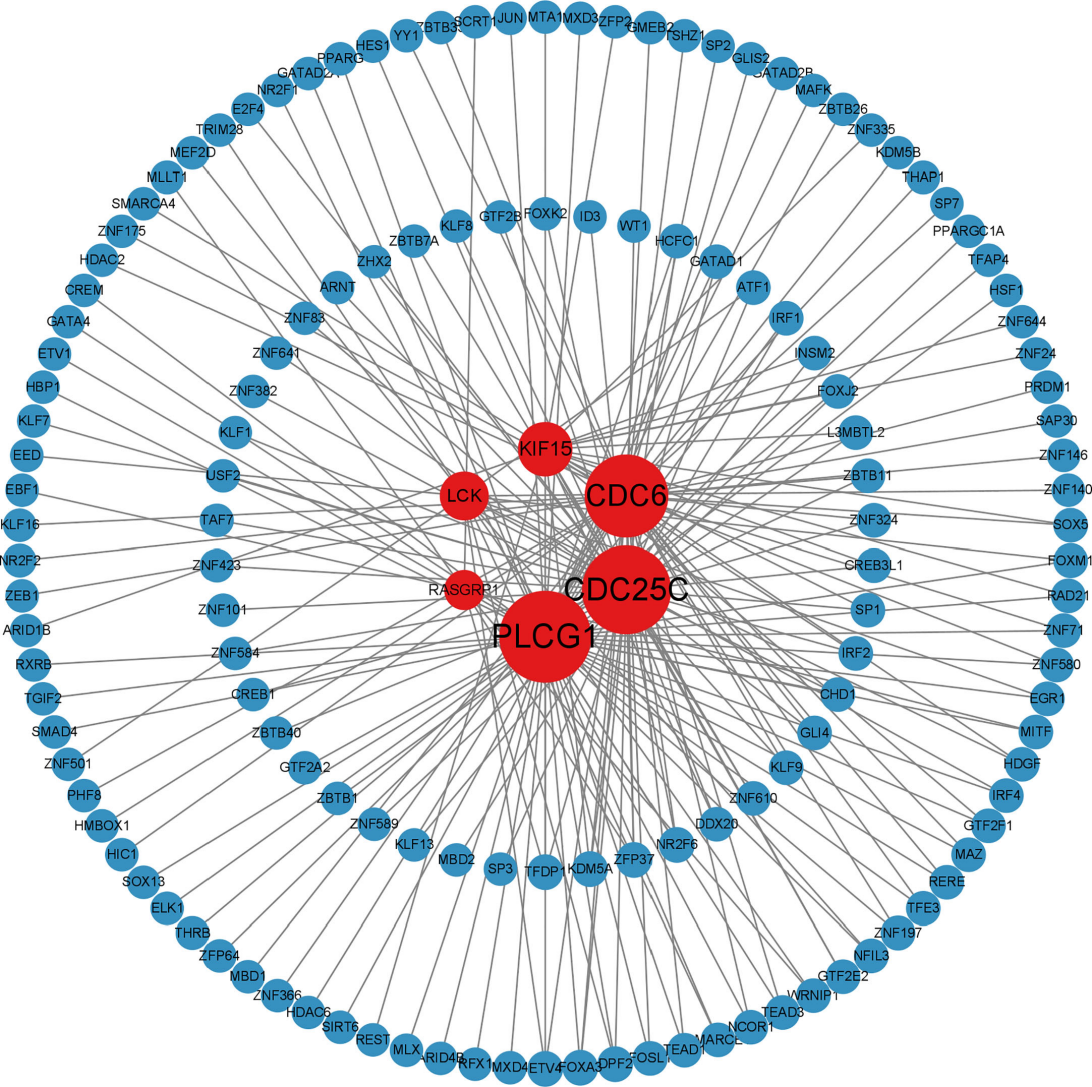
Differentially expressed TF-gene coregulatory network in SLE and COVID-19 interaction of TF with shared hub genes.

**Figure 8:**
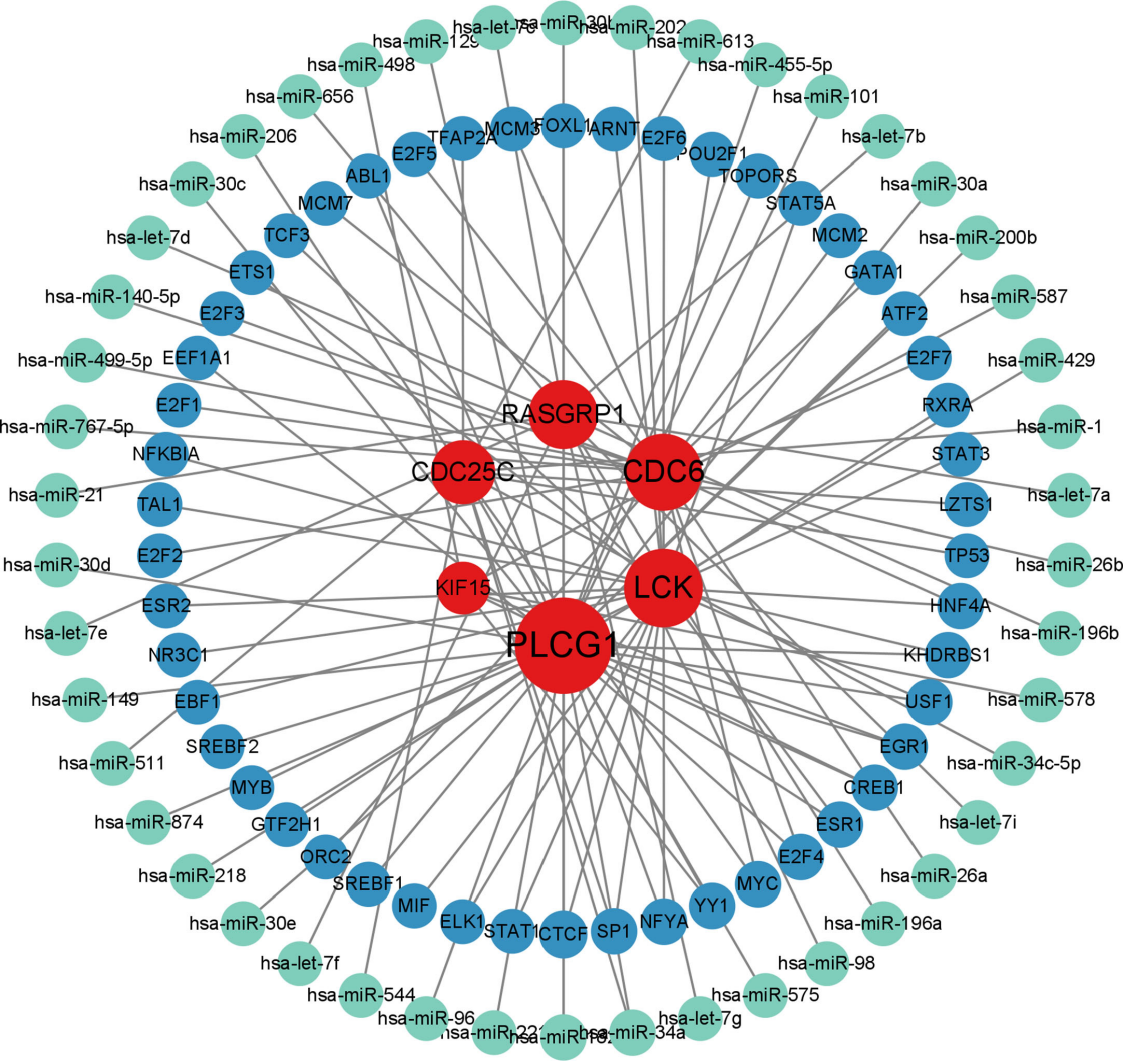
MiRNA-TF-gene co-regulatory network contributed to SLE and COVID-19 shared hub genes. (red nodes represent hub genes; blue nodes represent TFs; green nodes represent miRNAs; TFs: transcription factors).

The analysis of the interactions among integrative transcription factors (TF), miRNAs, and hub genes helps to unravel the biological processes of disease pathogenesis. In our study, we separately analyzed the interactions between the Networkanalyst platform containing the ENCODE (https://www.encodeproject.org/) database and the RegNetwork (http://www.regnetworkweb.org) database to construct the TF-gene interaction network and TF- miRNA co-regulatory networks. The regulatory networks were then imported into Cytoscape 3.7.2 for visualization and analysis. There were 140 TFs, 6 hub genes, and 193 edges were included in the TF-genes network (**Figure 7**), while a total of 111 edges, 46 miRNAs, and 45 TF genes interacted with the 6 hub genes in the TF-miRNA co-regulatory network (**Figure 8**).

### Validation of hub genes

**Figure 9:**
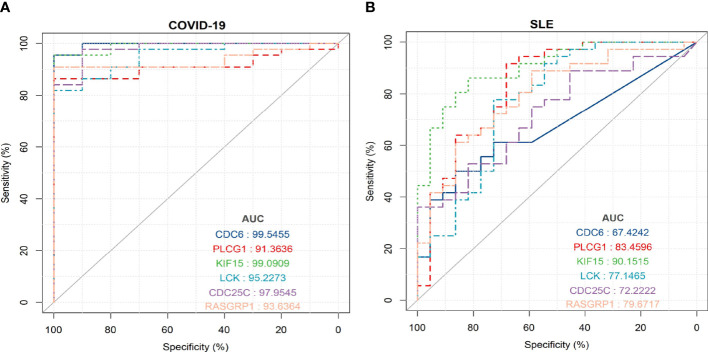
ROC curve for testing the diagnostic validity of shared hub genes in both datasets **(A)** ROC curve of shared hub genes in GSE171110 for diagnosis and efficacy verification. **(B)** ROC curve of shared hub genes in GSE22098 for diagnosis and efficacy verification.

To evaluate the diagnostic accuracy of the 6 shared hub genes in predicting disease-related outcomes, we performed ROC analyses for SLE and COVID-19, respectively. The area under the curve (AUC) values for 6 shared hub genes (*CDC6, PLCG1, KIF15, LCK, CDC25C*, and *RASGRP1*) in the SLE dataset to discriminate between patients and healthy controls were greater than 0.61 (**Figure 9B**), while those for the COVID-19 dataset were greater than 0.91 (**Figure 9A**). The ROC curves indicated that the 6 shared hub genes can helpfully predict the risk of COVID-19 with SLE. The results provided a rationale for targeting these hub genes in developing novel targeted therapies for SLE and COVID-19.

### Assessment and visualized analysis of the immune infiltration

**Figure 10:**
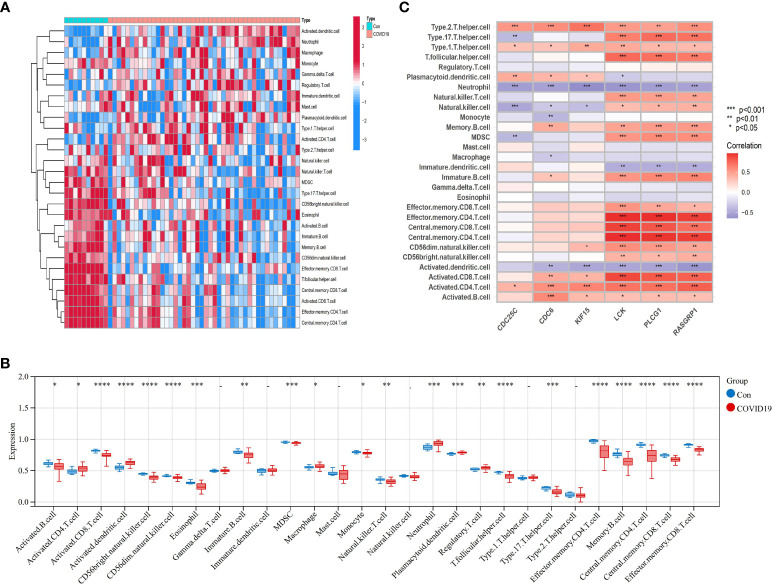
Immune cell infiltration analysis in COVID-19 dataset based on ssGSEA scores and an estimation of their association with shared hub genes **(A)** Hierarchical clustering of the distribution of the 28 immune cells in the GSE171110 (**Figure 10A**) samples. **(B)** Box plots of the proportions of different immune cells in the disease and healthy control, respectively in the GSE171110 (**Figure 10B**) samples. **(C)** Heatmap of correlation analysis between immune cell infiltration and 6 shared hub genes in the GSE171110 (**Figure 11C**) samples. The symbol * represents P < 0.05; ** represents P < 0.01; *** represents P < 0.001.

**Figure 11:**
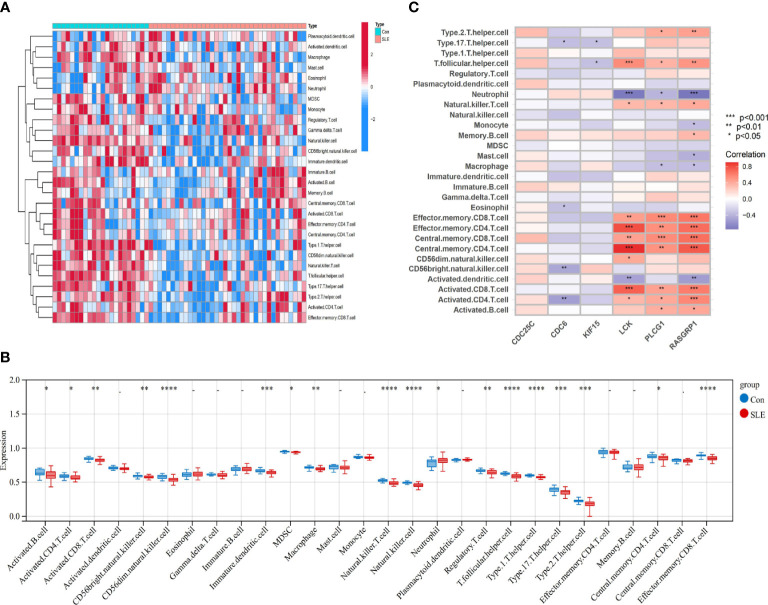
Immune cell infiltration analysis in SLE dataset based on ssGSEA scores and an estimation of their association with shared hub genes **(A)** Hierarchical clustering of the distribution of the 28 immune cells in the GSE22098 (**Figure 11A**) samples. **(B)** Box plots of the proportions of different immune cells in the disease and healthy control, respectively in the GSE22098 (**Figure 11B**) samples. **(C)** Heatmap of correlation analysis between immune cell infiltration and 6 shared hub genes in the GSE22098 (12CB) samples. The symbol * represents *P* < 0.05; ** represents *P* < 0.01; *** represents *P* < 0.001.

We used the one-sample GSEA (ssGSEA) algorithm to quantify the distribution (**Figures 10A**, **11A**) and relative proportions (**Figures 10B**, **11B**) of the relative infiltration levels of 28 immune cells in the GSE171110 and GSE22098 datasets. The correlation between immune cell infiltration and the shared hub genes were analyzed (**Figures 10C**, **11C**) to evaluate the differences in the immune micro-environment and characteristic pathways between the disease and healthy controls.

There were significant differences in the distribution and proportions of a variety of immune cells between COVID-19 and healthy controls, including activated CD4 T cells, macrophages, neutrophils, activated B cells, activated CD8 T cells, CD56 bright natural killer cells, eosinophils, etc. Analysis of the differences between SLE and healthy controls revealed a significantly higher infiltration of neutrophils in the peripheral blood of SLE. Immune cell ratios differed between the two groups and included: activated B cells, macrophages, natural killer cells, regulatory T cells, neutrophils, type 1 T helper cells, and type 17 T helper cells. As for the correlation analysis of 28 immune cells containing the 6 biomarkers showed that in the COVID-19 dataset, *LCK, PLCG1*, and *RASGRP1* were most significantly positively correlated with effector memory CD4 T cells, central memory CD4 T cells, and central memory CD8 T cells, respectively (*P* < 0.001). These biomarkers were the most significant negative correlation (*P* < 0.001) with activated dendritic cells and neutrophils (**Figure 10C**) conversely. In contrast, in the SLE dataset, *LCK*, *PLCG1*, and *RASGRP1* were positively correlated with activated CD8 T cells, effector memory CD4 T cells, central memory CD4 T cells, central memory CD8 T cells and effector memory CD8 T cells (*P* < 0.01), and negatively correlated with neutrophils (*P* < 0.05) (**Figure 11C**). These results provide further evidence for the critical role of these immune cells in the co-morbidity of COVID-19 and SLE.

## Discussion

Viral infection is an important environmental factor in the pathogenesis of SLE, and is strongly associated with disease onset and relapse (22). Previous studies have shown that Epstein-Barr virus (EBV), B19 virus (B19V), and human endogenous retroviruses (HERVs) were associated with the development of SLE (23–25). The viruses can induce a loss of immune tolerance, for one thing; For another, it may be related to the fact that long-term immunosuppressive therapy increases the risk of infection in patients with SLE (22).

The Coronavirus disease 2019 (COVID‐19) has progressed into a pandemic rapidly worldwide since it was first reported in December 2019. Not only can normal individuals suffer from COVID-19 infection, but patients with other chronic diseases are also affected, including SLE (26). SLE patients appear to be at higher risk of developing severe COVID-19 outcomes (27). Co-morbidity studies have demonstrated an increased risk of death and a poorer prognosis for SLE in combination with COVID-19 infection (28). SLE and COVID-19 have similar clinical phenotypes and molecular alterations, both of which can cause multiple organs and tissues, and their etiology is closely related to inflammatory pathways (29, 30). For example, both pathologies showed significantly dysregulated interferon (IFN) response and excessive inflammation. SARS-CoV-2 proteins block type I and type III IFN responses, further inducing monocyte and macrophage accumulation and activation, leading to massive IFN and pro-inflammatory cytokine production (31). On the other hand, SLE is characterized by activation of the IFN system, which results in increased expression of IFN-regulated genes, and IFN-I has been labeled as a biomarker and drug target of SLE (32). There may be a link between SLE and COVID-19. To the best of our knowledge, blood transcriptomic data have not been applied to analyze the value of diagnostic potential between SLE, COVID-19 and healthy controls. Therefore, we integrated independent datasets of the two diseases for bioinformatics and enrichment analysis and determined the relationship between SLE and COVID-19.

In our study, we first screened for 163 overlapped genes that may be involved in SLE and COVID‐19, which may be potential targets for SLE combined with COVID-19 infection treatment. Functional enrichment analysis of these common genes were primarily involved in immune cell activation functions, and KEGG signaling pathways mainly involved natural killer cell-mediated cytotoxic signaling pathways, nuclear factor-κB (NF-κB) signaling pathway, and Th17 cell differentiation. Previous blood transcriptomics studies have found that sustained activation of cytotoxic T cells and increased numbers of B cells are associated with COVID-19 infection and lung involvement, which is consistent with our findings (33, 34).

We finally identified 6 common hub candidate genes (*LCK, PLCG1, RASGRP1, CDC6, CDC25C*, and *KIF15*) and confirmed their diagnostic potency in two diseases by ROC curve. *PLCG1*, encoding the phospholipase C γ (PLCγ) 1 isoform, is a gene in the TCR cycle signaling pathway associated with mast cell activation in body-acquired immune function. *PLCG1* may act through the activation of mitogen-activated protein kinase and NF-κB signaling pathways (35). This is an important factor in the development of local histopathological changes in SLE. Therefore, targeted inhibition of this gene may be a new target for SLE therapy. Engagement of CD95 ligand (CD95) in response to calcium signaling *via* docking with *PLCG1* can induce the clustering of Th17 cells and exacerbate local histopathological changes in SLE (36). Therefore, targeted inhibition of this gene may be a novel therapeutic target for SLE.

Natalia, Cheshenko et al. have reported the importance of extracellular kinase function/phosphorylation events in viral infections, and that abnormal extracellular phosphorylation of PLCγ is involved in triggering COVID-19 (37).

*RASGRP1* is a Ras activating protein and belongs to the small G protein Ras guanine nucleotide exchange factors (GEFs) family. *RASGRP1* is mainly expressed in T cells and thymocytes, which can prevent virus infection and autoimmunity-related activated T cell proliferation (38). SARS-CoV-2 can damage the immune response of B and T cells by down-regulating the level of *RASGRP1* (39). *RASGRP1* maintains lymphocyte homeostasis and mice are defective. Mice deficient in *RASGRP1* may induce auto-reactive B cells disrupt immune tolerance through a T-cell mechanism, and are at increased risk of developing lymphoproliferative disorders characteristic of human SLE (40). It was observed that the expression level of *RASGRP1* in lymphocytes from SLE patients was decreased (41), which was consistent with the results of our analysis. In our study, the levels of *CDC6, CDC25C*, and *KIF15* were down-regulated in both the COVID-19 and SLE datasets, suggesting a better prognosis with these genes. The mechanisms of these potential oncogenes and cell cycle regulators genes in SLE and COVID-19 need to be further investigated.

In this work, enrichment analysis of the key modular genes were identified mainly in regulating T cell activation (GO: 0050863), natural killer cell-mediated immunity(GO: 0002228), T cell co-stimulation (GO: 0031295), T cell receptor signaling pathway, PD-L1 expression and PD-1 checkpoint pathway, NF-κB signaling pathway, and Th1 and Th2 cell differentiation. Genetic susceptibility to the development of SLE is associated with resistance to mechanisms that limit the activation of T cells and their differentiation to effector and memory cells (30). Abnormal activation of T cells is involved in the development of SLE and has been shown to mediate multi-system damages in SLE (42). COVID-19 is related to the activation of innate immunity. The increase of neutrophils, mononuclear phagocytes, and natural killer cells, and the decrease of T cells have been observed collecting in the affected lungs (43).

Programmed death (PD-1) is an important immunosuppressive molecule that modulates the immune system and promotes autoimmune tolerance by suppressing T-cell activity (44). The PD-1 axis is involved in regulating innate and adaptive immune sub-populations in SLE (45, 46). The PD-1 signaling pathway regulates the expression and activation of receptors on immune cells in the micro-environment, effectively blocking B-cell receptor signaling, and macrophages in SLE may also express PD-1 as a biomarker for their reduced ability to clear apoptotic cells (46).

It has been found that PD-1H knockout mice develop SLE-like manifestations. Meanwhile, activation of PD-1H with monoclonal antibodies reduced skin symptoms and decreased multiple autoimmune markers including autoantibodies, inflammatory cytokines and chemokines in lupus mice, suggesting that activation of PD-1H has a significant immunosuppressive effect (47). This study suggests that impaired PD-1H function is a key mechanism in the development of SLE, and PD-1H is expected to be a new target for SLE treatment (48). PD-L1 dysregulation is associated with COVID-19 pathogenesis (49). Patients with severe and critical COVID-19 infection exhibit dysregulated expression of the PD-1/PD-1 ligand (PD-L1) axis on the surface of innate immune cells and T cells, and circulating level of soluble PD-L1 is considered a prognostic biomarker and therapeutic target (50, 51).

The exertion of T cell function is dependent on the activation of the T cell receptor (TCR)-mediated signaling pathway. The corresponding transcription factors are finally activated, which regulate the expression of effector protein molecules and complete the activation of T cells in COVID-19 (52, 53). Activation of the *LCK* gene is a key part of TCR signaling initiation (54).

The NF-κB signaling pathway is a typical pro-inflammatory pathway responsible for up-regulating the expression of inflammatory cytokines, chemokines, etc. It is a vital pathway that causes cytokine storm and plays an important role in disease progression and exacerbation in COVID-19 (55). Multiple cytokines and miRNAs participate in regulating classical and non-classical NF-κB signaling pathways in the occurrence and development of SLE (56–58)

Studies have reported that after SARS-CoV-2 infection, circulating B, T, NK cells, monocytes, the eosinophils/basophils decreased in severe patients, and the proportion of neutrophils increased significantly. Cytotoxic T lymphocytes (CTLs) are involved in the down-regulation of immune activation through their ability to kill T cells, NK cells, and antigen-presenting cells (59, 60). The bronchoalveolar lavage fluid of patients with severe COVID-9 contains a large number of macrophages and neutrophils derived from pro-inflammatory monocytes, thereby promoting local inflammation (61).

Neutrophils play a crucial role in innate immunity, serving as the primary line of defense against microbial infections and helping to maintain the stability of the body’s internal environment. During acute inflammation, neutrophils can rapidly reach the site of infection through chemotaxis, phagocytosis, and degranulation, effectively combating pathogens. Additionally, neutrophils play a regulatory role in adaptive immunity (62). One of the bactericidal mechanisms utilized by neutrophils is the release of neutrophil extracellular traps (NETs), a reticular structure comprised of histones and double-stranded deoxyribonucleic acid (dsDNA), which can immobilize and kill pathogenic microorganisms, a structure known as neutrophil extracellular traps (NETs) and the process by which they are generated is known as NETosis (63). Neutrophils are involved in autoimmune diseases. Some autoantibodies promote NET release through NETosis and molecular exocytosis (MPO and double-stranded DNA are autoantigens in systemic autoimmune diseases) (64). Interestingly, studies have confirmed the pathogenic role of such neutrophil-derived NETs in a variety of inflammatory states including COVID-19 infection and SLE (65–67). In SLE patients, neutrophils exhibit phenotypic and functional abnormalities such as failure of C1q/calreticulin and CD91-mediated apoptotic pathways to clear phagocytic defects, increased aggregation of abnormal oxidative activity, and increased numbers of circulating low-density granulocytes (LDGS) (68). This cycle of NETosis and autoantibody production perpetuates antigen release through NETs and autoantibody production.

Autoreactive B cell activation drives human SLE initiation and progression with subsequent breakdown of B cell tolerance followed by the production of large numbers of auto-antibodies (69).

Aberrant regulation of innate (including macrophages, dendritic cells, neutrophils, and NK cells) and adaptive immune (including T and B cells) responses are fundamental features of SLE (70). It has been demonstrated that the number and cytotoxic function of peripheral NK cells is reduced, neutrophils are dysregulated, reactive oxygen species generated during phagocytosis are reduced, and inflammatory responses are enhanced (71, 72). The results of our immune in filtration analysis study are consistent with previous analyses.

There are limitations regarding the current study that need to be elucidated. Firstly, the sample size of the compliant datasets and gene sets is small. Secondly, no validation has been performed on large samples at the single cell or protein level. This study provides new diagnostic biomarkers for SLE and covid-19 by integrating bioinformatics approaches, and immune infiltration study models, and there is a need to confirm the results of this work through prospective experiments.

## Conclusion

We first provided bioinformatic evidence that SLE and COVID-19 pathogenesis may be linked, and identified 6 common genes as diagnostic biomarkers for SLE associated with COVID-19. These genes were mainly enriched in multiple immune cell activation, cell cycle, TCR signaling pathway, PD-L1 expression and PD-1 checkpoint pathway, and NF-κB signaling pathway, and are closely related to the immune cell ratios. This study offers new research prospects for the diagnosis and treatment of SLE and COVID-19.

## Data availability statement

The original contributions presented in the study are publicly available. This data can be found here: https://www.ncbi.nlm.nih.gov/geo/ under the accession numbers GSE22098 and GSE171110.

## Author contributions

Conception and design: HZ, YZ, and XL. Data curation: all authors. Formal analysis: HZ, XL and XH. Investigation: all authors. Methodology: all authors. Project administration: YZ, HP, and XH. Resources: all authors. Data analysis: ZY,HP, and XH. Supervision: HZ, XL, and YZ. Writing—original draft: HZ and YZ. Writing—review and editing: HZ, ZY, and XH. All authors contributed to the article and approved the submitted version.
